# A Novel Active Learning Framework for Cross-Subject Human Activity Recognition from Surface Electromyography

**DOI:** 10.3390/s24185949

**Published:** 2024-09-13

**Authors:** Zhen Ding, Tao Hu, Yanlong Li, Longfei Li, Qi Li, Pengyu Jin, Chunzhi Yi

**Affiliations:** 1 College of Computer and Control Engineering, Northeast Forestry University, Harbin 150040, China; 2 School of Mechatronics Engineering, Harbin Institute of Technology, Harbin 150001, China; 22s136122@stu.hit.edu.cn (T.H.); 13703536875@163.com (Y.L.); 17762377869@163.com (L.L.); 22s136121@stu.hit.edu.cn (Q.L.); 23s136412@stu.hit.edu.cn (P.J.); 3 School of Medicine and Health, Harbin Institute of Technology, Harbin 150001, China; chunzhiyi@hit.edu.cn

**Keywords:** wearable sensors, human activity recognition, cross-subject issue, relation network, classifier discrepancy, surface electromyography signals

## Abstract

Wearable sensor-based human activity recognition (HAR) methods hold considerable promise for upper-level control in exoskeleton systems. However, such methods tend to overlook the critical role of data quality and still encounter challenges in cross-subject adaptation. To address this, we propose an active learning framework that integrates the relation network architecture with data sampling techniques. Initially, target data are used to fine tune two auxiliary classifiers of the pre-trained model, thereby establishing subject-specific classification boundaries. Subsequently, we assess the significance of the target data based on classifier discrepancy and partition the data into sample and template sets. Finally, the sampled data and a category clustering algorithm are employed to tune model parameters and optimize template data distribution, respectively. This approach facilitates the adaptation of the model to the target subject, enhancing both accuracy and generalizability. To evaluate the effectiveness of the proposed adaptation framework, we conducted evaluation experiments on a public dataset and a self-constructed electromyography (EMG) dataset. Experimental results demonstrate that our method outperforms the compared methods across all three statistical metrics. Furthermore, ablation experiments highlight the necessity of data screening. Our work underscores the practical feasibility of implementing user-independent HAR methods in exoskeleton control systems.

## 1. Introduction

In recent years, wearable exoskeleton robots have emerged as a promising technology for the augmentation of human capabilities and assisting patients with rehabilitation [1,2,3,4]. Their applications span various fields, including industrial [5], medical [6], and military [7] domains. The accurate recognition of human activities forms the cornerstone of effective exoskeleton control [8]. With advancements in wearable sensors, human activity recognition (HAR) methods [9,10], which leverage diverse sensing information, are increasingly emerging. Although recent methods have shown promising performance on multiple open-source datasets, the adaptation of these HAR methods for new users remains a challenge [11,12,13].

The use of surface electromyography (sEMG) signals for motion intention recognition shows unique advantages and great prospects in the field of exoskeletons [14,15,16]. However, the performance of HAR methods significantly degrades [17,18] when applied to new individuals due to factors such as individual variability in sEMG signals, sensor electrode shifts, and limb position shifts. While deep learning techniques have the ability to automatically learn data distributions, the cross-individual issue persists. Fine tuning is a commonly used fully supervised solution that involves adjusting and updating model parameters using a large-scale, target-labeled dataset to improve performance on the target individual [19]. However, fine-tuned models often suffer from catastrophic forgetting [20], and the collection of large-scale labeled datasets is both time-consuming and labor-intensive.

Convolutional neural networks (CNNs) have gained significant traction in HAR recently [21,22,23]. Atzori et al. [24] demonstrated that even CNN with a simple architecture can outperform traditional machine learning methods in classification tasks. However, directly applying CNNs to address cross-subject issues has shown limited effectiveness. Therefore, some researchers have attempted to improve the generalization ability of algorithms by increasing model complexity [25] or by choosing model architectures adapted to specific signals [26]. Ali et al. [27] introduced a pioneering single hybrid model, amalgamating the merits of both CNNs and transformer neural networks. The model incorporates a CNN block to capture local dependencies and a transformer block to capture long-range global dependencies, thereby enhancing the model’s feature-capturing capability. Rahimian et al. [28] introduced a hybrid deep learning architecture for multi-channel sEMG signals. This architecture combines a long short-term memory path for temporal feature extraction with a CNN path for spatial feature extraction. Although increasing model complexity can alleviate the performance degradation of prediction algorithms, it fails to fundamentally address the challenge of inconsistent data distribution across different domains when dealing with novel individuals.

Another commonly used approach is domain adaptation (DA), which aims to reduce the disparities between data distributions in different domains [29]. DA is classified into two types, namely supervised domain adaptation (SDA) and unsupervised domain adaptation (UDA), depending on the availability of labeled data in the target domain. SDA algorithms use the labeled target-domain data to adjust model parameters directly, thereby realizing the adaptive process of feature space and the classification hyperplane. Twin architectures have been frequently utilized in numerous studies [30,31], likely due to the advantageous feature of parameter sharing. For instance, Bao et al. [32] developed a dual-stream CNN for the extraction of domain-invariant features. By introducing an additional domain discrepancy loss, the distribution mismatches between the two domains can be effectively minimized. UDA provides an effective approach by aiming to align data features from disparate domains into a unified feature space, thereby minimizing discrepancies. Two prevalent categories of UDA approaches are extensively employed. The first is loss-based UDA, which minimizes domain differences through the effective utilization of multiple loss functions [33,34]. The second is adversarial-based UDA, which encourages classifiers and feature extractors to perform adversarial training [35,36]. However, UDA methods typically rely on data-driven techniques for knowledge transfer, and they also face challenges of unknown data quality and distribution in the target domain. This poses a significant hurdle for models striving to achieve both high accuracy and fast adaptation speed.

These DA approaches primarily focus on the model’s adaptation process, often overlooking the importance of target data with respect to model performance. The variance in data distribution directly influences the adaptation strategies employed. When the target-domain data are near the classification hyperplane, they exhibits high uncertainty, posing challenges for accurate classification by the model. To address this issue, flexibly adjusting the data feature space enables the model to effectively adapt to variations in domain distribution. Conversely, target data deviating significantly from the classification hyperplane tend to have higher confidence and allow the model to classify them accurately. In such a scenario, a stable conditioning strategy may be more suitable to maintain the model’s robustness. Consequently, considering the significance of target data distribution and employing appropriate adaptation strategies become essential in optimizing the model’s overall performance.

This paper alleviates the cross-subject issue of HAR from the perspective of feature space and self-adaptation. First, a twin relation network architecture is introduced to extract the core features of dual-input electromyography (EMG) samples. In the dual-input form, relation features are introduced on the basis of the feature space of source-domain data, enabling a comparison of similarity between the input sample and the template data to predict the motion category. Concurrently, the framework conducts differential sampling of target data based on classifier discrepancy. The sampled data are then utilized to optimize the template data distribution and fine tune model parameters, significantly improving the model’s cross-subject performance and stability. In this study, we conducted a series of experiments using both a public dataset and a self-built dataset. Our experimental findings reveal that our method performs best on all three statistical metrics among the compared methods. Furthermore, ablation experiments underscore the importance of data screening. The contributions of this study are summarized as follows:

(1) A relation network architecture that incorporates a similarity feature space alongside the original feature space is introduced. By comparing the similarity between the input sample and the template data for motion prediction, the classification performance is significantly improved.
(2) A novel bidirectional optimization strategy is proposed, aiming to enhance the model’s cross-subject performance. This strategy involves adjusting model parameters backward and optimizing the distribution of template data forward.
(3) A data importance screening strategy is proposed that utilizes classifier discrepancy to measure the target data distribution. This strategy helps identify the significance of different data samples and facilitates model adaptation.

The remainder of this paper is structured as follows. Section 2 describes the active learning framework, detailing the relation network architecture, bidirectional optimization strategy, and data sampling process. Section 3 introduces the experimental datasets, the design of the self-built dataset collection, and the experimental validation results. Section 4 discusses the experimental results in detail, followed by a conclusion in Section 5.

## 2. Method

### 2.1. Problem Definition

For cross-subject HAR, we assume the following conditions. Given a labeled source domain ($D_{L}=\left\{\left(x_{l},y_{l}\right)\right\}$) and an unlabeled target domain ($D_{U}=\left\{x_{u}\right\}$), consider a model ($G_{\phi }\left(\cdot \right)$*$F_{\theta }\left(\cdot \right)$, $F_{\theta 1}\left(\cdot \right)$, and $F_{\theta 2}\left(\cdot \right)$) that has undergone pre-training with the source-domain dataset. Due to individual differences, the joint distribution of source and target domains varies. Moreover, the absence of distribution and quality information in unlabeled target data poses a significant obstacle to model adaptation.

This work aims to leverage the sampling function (S(·)) to acquire high-quality target-domain data. Subsequently, we utilize the tagged target-domain data to adjust the model bidirectionally, thereby improving the model’s individual adaptability. For clarity, Table 1 lists the notations used in this study.

### 2.2. Framework Model

To address the challenges of recognizing human activity across different subjects, we propose an active learning framework centered on strategic data screening. The framework initially uses unlabeled target sEMG data to fine tune the two auxiliary classification boundaries of the pre-trained model. This is followed by the sampling and labeling of the target data. Finally, the target-domain template and the fine-tuning datasets are updated. The framework employs forward distribution adjustment and backward parameter update optimization strategies to facilitate the self-adaptation of the predictive model.

#### 2.2.1. Overview of the Framework

**Figure 1:**
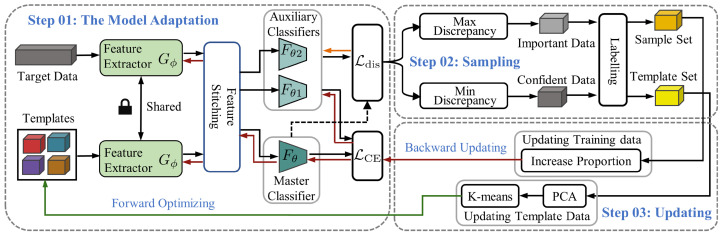
Overview of the active learning framework. The flow chart consists of three steps. The black arrows indicate the forward process. The orange arrows indicate the process of adjusting the auxiliary classification boundaries. The deep-red arrows indicate the model update process. The green arrow indicates the template data optimization process.

Figure 1 illustrates the architecture of the active learning HAR model. The proposed framework, based on the relation network, can be structured into the following three integral components: model adaptation, data sampling, and updating.

1. **Motion Prediction and Model Adaptation with a Dual Strategy:** The forward algorithm predicts motion intention based on target data and template data. The dual adaptation strategy tunes model parameters and feature distribution through backward propagation and forward template distribution adaptation, respectively.
2. **Sampling Data Based on Classifier Discrepancy:** Data sampling focuses on obtaining high-quality samples from the target domain for adaptation. It classifies target sEMG data into “important data”, located near the classification hyperplane, and “confident data”, positioned further away, using a discrepancy metric. The adaptability of this study relies on independently processing these two distinct types of data.
3. **Updating the Template Data and Training Data:** Adjustment strategies diverge based on data distribution disparities. Our proposed method employs a dual strategy for fine tuning, leveraging both template and training data buffers. In this step, the algorithm screens these two distinct data types to participate in the optimization process, improving the cross-subject performance of the model.

#### 2.2.2. CNN-Based Relation Network for Model Prediction

In this paper, a twin network architecture generates the final classification outcome by calculating relation scores between the input sample and each class template. This network consists of a feature extractor ($G_{\phi }\left(\cdot \right)$) and three classifiers (one main classifier ($F_{\theta }\left(\cdot \right)$) and two auxiliary classifiers ($F_{\theta 1}\left(\cdot \right)$ and $F_{\theta 2}\left(\cdot \right)$)).

**Figure 2:**
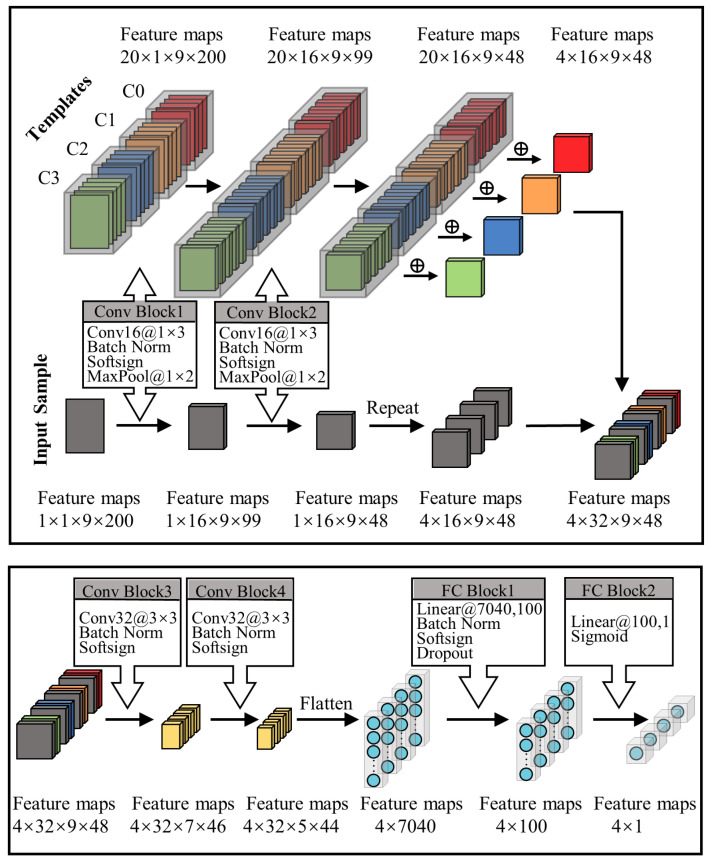
Relation network architecture and information on the model parameters. (**Top**): Feature map information of the input sample and templates after the feature extractor and feature stitching operation based on our EMG dataset. (**Bottom**): Feature map information of the combined features after the classifier based on our EMG dataset.

The feature extractor is designed to derive four relation matrices, each containing features from both the input data and template data across all classes. The feature extractor comprises two convolution blocks (Conv blocks). Each block consists of a sequence comprising a convolutional layer, followed by batch normalization and a Softsign activation, and concludes with a max-pooling layer for subsampling. The convolutional layers utilize a kernel size of 1 × 3 with 16 filters and a stride of 1, while the max-pooling layers employ a size of 1 × 2. Based on the parameter-sharing technique, the Conv blocks process the input data and four sets of template data independently (each set representing a movement pattern and containing five sample data). As depicted in Figure 2, the features of both input data and template data are replicated and summed, then combined to obtain four relation matrices.

The classifier is tasked with computing the similarity between the input data and the class-specific template features within each relation matrix, as shown in Figure 2. It identifies the maximum similarity as a predictor of motion. The classifier comprises two Conv blocks and two fully connected blocks (FC blocks). Each Conv block has a convolutional layer, a batch normalization layer, and a Softsign layer. The convolutional layer, with a kernel size of 3 and stride of 1, is used to capture information on each relation matrix. The first FC block integrates a fully connected layer with 100 hidden units, followed by a batch normalization layer, a Softsign activation layer, and a dropout layer. The subsequent FC block simplifies this structure, containing only a single hidden unit in its fully connected layer and a Sigmoid activation layer for output. The proposed method assigns a value of 4 to the batch dimension, representing four relation matrices. By independently processing the four relation matrices, the classifier outputs four scalars. These scalars range from 0 to 1 and represent the similarities between the query sample and the templates, termed relation scores.

### 2.3. Model Adaptation Based on a Backward Strategy

The backward optimization strategy utilizes unlabeled target data and sampled data to adjust the auxiliary model parameters and main model parameters, respectively, thereby improving the model’s cross-subject performance. The process is based on the pre-trained model and is guided by the following two optimization objectives: improving motion intent prediction accuracy and refining decision boundaries for auxiliary classifiers. The methodologies for achieving these objectives are outlined as follows.

The feature extractor and main classifier can be considered a unified entity ($G_{\phi }\left(\cdot \right)$*$F_{\theta }\left(\cdot \right)$) and are trained using cross-entropy loss based on the truth label ($y_{s}$) of the input sample ($x_{s}$). The training objective is expressed as follows:

(1) $$\min_{G_{\phi },F_{\theta }}L_{CE}\left(G_{\phi },F_{\theta }\right)$$

(2) $$L_{CE}={-E}_{\left(x_{s},y_{s}\right)\in S}\sum_{c=1}^{C}1_{\left[c=y_{s}\right]}\;log\;r^{c}\left(\left.y_{s}\right|x_{s}\right)$$

where the other inputs of the model are the template samples from the template set (*T*). *C* denotes the total number of classes. $r^{c}\left(\left.y_{s}\right|x_{s}\right)$ is the output of the classifier network and denotes a relation score of class *c* for sample $x_{s}$. For function $1_{\left[a\right]}$, it equals 1 when predicate *a* is true and 0 otherwise.

The two auxiliary classifiers are trained using both cross-entropy loss and discrepancy loss to achieve tighter decision boundaries for evaluation data. These auxiliary classifiers share the same network as the main classifier. The training objective of the auxiliary classifiers is formulated as follows:

(3) $$\begin{array}{ccc} \underset{F_{\theta 1},F_{\theta 2}}{min} & {\;L}_{CE}\left(G_{\phi },F_{\theta 1}\right)+L_{CE}\left(G_{\phi },F_{\theta 2}\right) & -L_{dis}\left(G_{\phi },F_{\theta 1},F_{\theta 2}\right) \end{array}$$

(4) $$L_{dis}=E_{x_{u}\in D_{U}}\left[d\left(r_{1},r\right)+d\left(r_{2},r\right)+d\left(r_{1},r_{2}\right)\right]$$

(5) $$r_{1}\left(x_{t}\right)=F_{\theta 1}\left(M\left(G_{\phi }\left(x_{t}\right),G_{\phi }\left(x_{u}\right)\right)\right)\in R^{C}$$

(6) $$r_{2}\left(x_{t}\right)=F_{\theta 2}\left(M\left(G_{\phi }\left(x_{t}\right),G_{\phi }\left(x_{u}\right)\right)\right)\in R^{C}$$

where $L_{dis}$ represents the sum of the discrepancy values between the outputs of all classifiers. The symbol *d* represents the discrepancy distance, which, in this study, is defined by the Manhattan distance as follows:

(7) $$d\left(r_{1},r_{2}\right)=\frac{1}{C}\sum_{c=1}^{C}\;\left|r_{1}^{c}-r_{2}^{c}\right|$$

where the $r_{1}^{c}$ and $r_{2}^{c}$ represent the relation scores of $r_{1}$ and $r_{2}$ for class *c*, respectively.

It is crucial to emphasize that the performance of the auxiliary classifiers is not a primary concern. Their main role lies in refining decision boundaries for sample acquisition rather than accurately classifying the task. Additionally, the training of the auxiliary classifiers does not impact the main classifier and feature extractor.

### 2.4. Model Adaptation Based on a Forward Strategy

The backward strategy facilitates adaptive learning by adjusting the network’s parameters, while the forward strategy achieves adaptation by modifying the distribution of input template data. In this study, motion prediction is achieved by the relation network, which calculates the similarity between input data and the template data of each class in the feature space. Consequently, altering the distribution of template data can directly impact the classification outcome.

To obtain more diverse template samples, principal component analysis (PCA) is first employed to reduce the feature of template data to three dimensions. Subsequently, the K-means clustering algorithm is used to categorize the template data from each class into five distinct clusters. The central samples of each cluster are selected to assemble a template group. This process of updating the template data allows the model to capture individualized features, even if the sEMG samples are from the same class.

### 2.5. Data Sampling for a Dual Strategy

**Figure 3:**
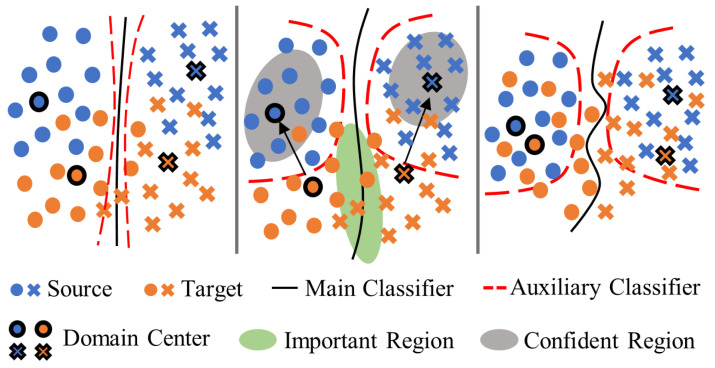
Illustration of auxiliary classifier training and data distribution adjustment. (**Left**): All classifiers are similar in the initial training stages. (**Middle**): Two tight auxiliary classification boundaries are obtained after adjusting with the discrepancy loss. (**Right**): The data distribution after fine tuning the model using sampled data.

The quality of data directly affects the performance and stability of model adaptation. Samples close to decision boundaries, characterized by higher uncertainty, indicate a greater potential for loss and are crucial for improving model performance. Conversely, samples that are distant from decision boundaries exhibit more general features and play a vital role in updating the class templates within the relation model’s input and enhancing the stability of the model. After training the auxiliary layers, two tight decision boundaries can be obtained, as shown in Figure 3. Unlabeled samples located between the two decision boundaries (we refer to these samples as “importance data”) are characterized by being far from the data distribution and difficult to train, while samples located outside the two decision boundaries (we refer to these samples as “confident data”) have the characteristics of being well characterized and close to the center of the domain. This study proposes a data screening method relying on two auxiliary classifiers and their tight decision boundaries to select training data for forward and backward strategies. Considering the distribution differences between different domains, the sample acquisition function (S(·)) can be written as follows:

(8) $$S\left(x_{u}\right)=\left|D\left(x_{u}\right)-\frac{1}{\left|D_{L}\right|}\sum_{x_{l}\in D_{L}}\;D\left(x_{l}\right)\right|,\;for\;\;x_{u}\in D_{U}$$

In this study, S(·) is used to sample the data by comparing the average output discrepancy values of the samples in the target domain. A large function value indicates that the data are far from the data distribution, while a small value indicates that the data are close to the center of the domain. The discrepancy function ($D\left(x\right)=d\left(r_{1},r_{2}\right)+d\left(r,r_{1}\right)+d\left(r,r_{2}\right)$) is employed to make the best of the discrepancy among all classifiers. During the data sampling process, by calculating the average classifier discrepancy of each sample in the target domain, we select the first $n_{1}$ samples with the maximum discrepancy value to be labeled to constitute the sample set (*S*) and the last $n_{2}$ samples with the minimum discrepancy value to be labeled to constitute the template set *T*, as illustrated in Algorithm 1.

The backward strategy improves model performance by boosting the participation of “important data” in the parameter update process. Simultaneously, the forward strategy refines the input template data distribution by focusing on selecting representative data that fall within the “confident data” category, thereby enhancing the model’s stability.
**Algorithm 1:** Data Sampling
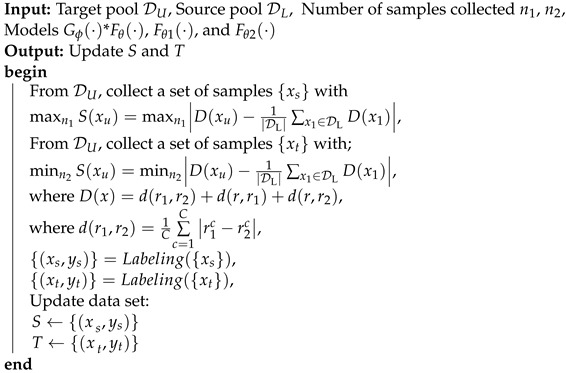


## 3. Experiment

### 3.1. Experimental Dataset

In this paper, to validate the effectiveness and superiority of the proposed framework, we use a public dataset and a self-constructed dataset for testing and evaluation. Both of them are EMG-based datasets. Below, we introduce these two datasets and provide a detailed description of the process for creating the self-constructed EMG dataset.

**(1)**  **The Public Dataset:** The ENABL3S dataset is a standardized bilateral lower-limb movement EMG dataset comprising sEMG data from seven male and three female subjects during daily activities [37]. During data collection, each subject was required to perform two types of experiments, with each type repeated 25 times. Both experiments included the same activities, namely sitting, standing, ascending/descending a ramp, ascending/descending a staircase, and level ground walking. The only difference between the two experiments was the sequence of these activities. Subjects were instructed to switch between activities at their chosen speed and to take regular breaks to prevent muscle fatigue. Notably, this dataset includes the transition phases between different activities, effectively simulating real-life movement scenarios. Surface EMG electrodes were placed on the same seven muscles of both legs of the subjects and sampled at a frequency of 1 kHz.
**(2)**  **The Self-Constructed EMG Dataset:** Surface EMG signals were collected from subjects during walking to construct our EMG dataset. Ten healthy participants (labeled as Subject 1 to Subject 10, aged 22 to 29) without any history of lower limb or neuromuscular disease were recruited for this experiment. Before data collection, informed consent was acquired from all participants, and the experimental procedures received approval from the Chinese Ethics Committee of Registering Clinical Trials.

**Figure 4:**
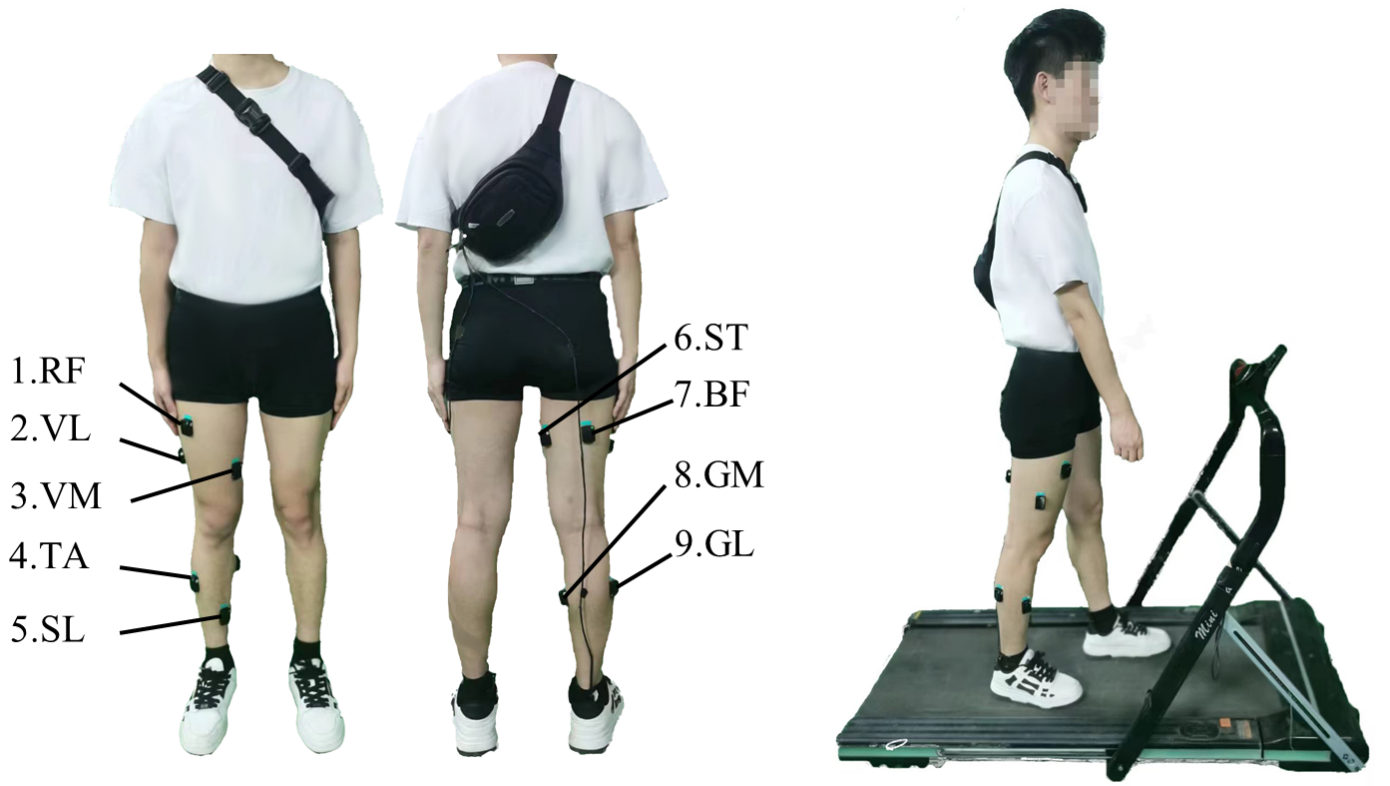
Experimental scheme. (**Left**): Location of the EMG sensors. (**Right**): Example of a walking experiment.

The Delsys Trigno system was employed for sEMG signal acquisition. As shown in Figure 4, electrode sensors were attached to nine lower limb muscles, including the rectus femoris (RF), vastus lateralis (VL), vastus medialis (VM), tibialis anterior (TA), soleus (SL), semitendinosus (ST), biceps femoris (BF), gastrocnemius medial head (GM), and gastrocnemius lateral head (GL), with a sampling frequency 1111.11 Hz. These muscles have been identified as valuable for lower limb movement recognition [38]. Foot pressure sensors were used to capture pressure signals at different positions of the foot to accurately identify different gait phases, including heel strike, foot flat, heel off, and toe off [39]. These sensors were positioned on the heel and the first metatarsal, operating at a sampling frequency of 500 Hz. Note that the EMG sensors and the foot pressure sensors were synchronized to acquire signals.

Participants were instructed to maintain a steady speed of 5 km/h while walking on a treadmill, as shown in Figure 4. Each trial lasted approximately 8 min, but only the central 5-min portion of sEMG data from each trial was taken as the final experimental data. To minimize muscle fatigue, three trials were conducted for each participant, with adequate rest in between. Motion artifacts were eliminated using a three-order Butterworth filter (20 Hz), while high-frequency noise was filtered out using a low-pass filter (450 Hz). Additionally, the surface EMG of each channel was normalized by dividing it by the peak value of the corresponding muscle during normal walking.

**(3)**  **Data Segmentation:** In this paper, data segmentation is performed using a sliding window approach with a length of 200ms and an increment of 25 ms. Consequently, in our experiments, the size of the sEMG matrix (1 × N × L) in the ENABL3S dataset was 1 × 14 × 200, whereas in our EMG dataset, the size of the sEMG matrix is 1 × 9 × 200.

### 3.2. Experimental Setup

In this study, we adopted a leave-one-out cross-validation approach to evaluate the effectiveness of our HAR model. Specifically, for each validation round, one subject was designated as the testing target, whereas the data from the remaining subjects were used for pre-training of the model. The network was trained and tested using the Pytorch backend. The relation network was trained for 100 epochs using the adaptive moment estimation (ADAM) optimizer with a small batch size of 64. An exponential decay strategy was utilized to dynamically adjust the learning rate, starting from an initial rate of 0.5 and decaying by 0.95 every two epochs. Additionally, the dropout rate was set to 30%.

### 3.3. Model Evaluation

**(1)**  **Evaluation of Effect of the Number of Templates on Performance:** To select the appropriate number of templates for subsequent experiments, we compared the accuracy and average testing time for five subjects in the target test set for 1 shot, 5 shots, and 10 shots. These five subjects were randomly selected and consecutive.
**(2)**  **Evaluation Across Different Methods:** We selected several mainstream methods designed to address the cross-subject issue for comparative experiments. These methods use our framework to mitigate the influence of other variables on performance. Below, we provide a brief introduction to these selected methods.
  1. Relation Network for Few-Shot Learning [40] (RN-FSL): RN-FSL serves as an innovative framework for few-shot learning, with its primary objective being the training of a deep distance metric for accurate classification of images belonging to new classes.
  2. Maximum Classifier Discrepancy for Unsupervised Domain Adaptation [36] (MCD): MCD is an unsupervised domain-adaptive method aimed at aligning the distributions of the source and target domains by using task-specific decision boundaries.
  3. Maximum Classifier Discrepancy for Active Learning [41] (MCDAL): MCDAL presents a novel active learning framework that utilizes the prediction discrepancies among multiple classifiers to guide sample acquisition.
**(3)**  **Evaluation within the Framework:** As outlined in Section 2.5 and Figure 1, the screening of template samples and training samples is indispensable, since they play a crucial role in model transfer performance. Thus, we performed two ablation experiments to evaluate the necessity of screening template samples and training samples. First, for template samples, we compared the model’s accuracy on target subjects when using the following three types of samples as templates: “important data”, random data, and “confident data”. Secondly, for training samples, we compared the effects of “important data” and random sampling data on the model’s classification accuracy. These two sets of experiments are validated using the ENABL3S dataset and our EMG dataset.
**(4)**  **Evaluation Metrics:** To comprehensively quantify the recognition performance of our method, the following statistical evaluation metrics are introduced for precise performance evaluation: (1) accuracy, (2) precision, and (3) recall. These metrics are formulated as follows:
  (9) $$\begin{array}{cc} Accuracy & =\frac{TP+TN}{TP+FN+FP+TN}\times 100\%\\ Precision & =\frac{TP}{TP+FP}\times 100\%\\ Recall & =\frac{TP}{FN+TP}\times 100\% \end{array}$$
  where TP and TN represent true positive and true negative, respectively, while FP and FN represent false positive and false negative, respectively. The testing time serves as a metric to evaluate the computational overhead of the model, defined as the duration from input data ingestion to the generation of model outputs.

## 4. Experimental Results

**(1)**  **Experiment on the Number of Templates:** Table 2 presents the experimental results of template quantity on the ENABL3S dataset and our EMG dataset. In the ENABL3S dataset, the average classification accuracy for the seven-way, one-shot scenario is only 53.55%. However, the seven-way seven-shot and 7-way 10-shot scenarios show significant improvements, with average classification accuracies reaching 74.22% and 77.74%, respectively. The average testing times for these scenarios are 3.29 ms, 4.95 ms, and 7.07 ms, respectively.

For our EMG dataset, the highest classification accuracies among the five subjects in the 4-way, 1-shot; 4-way, 5-shot; and 4-way, 10-shot scenarios are 73.55%, 81.17%, and 85.06%, respectively, with average testing times of 1.94 ms, 2.87 ms, and 4.98 ms, respectively. As seen in Table 2, both subject-specific accuracies and average testing times increase as the number of templates per class increases. Therefore, this subsection selects the five-shot scenario for subsequent study by evaluating the influence of template quantity on both model performance and time cost.

**Figure 5:**
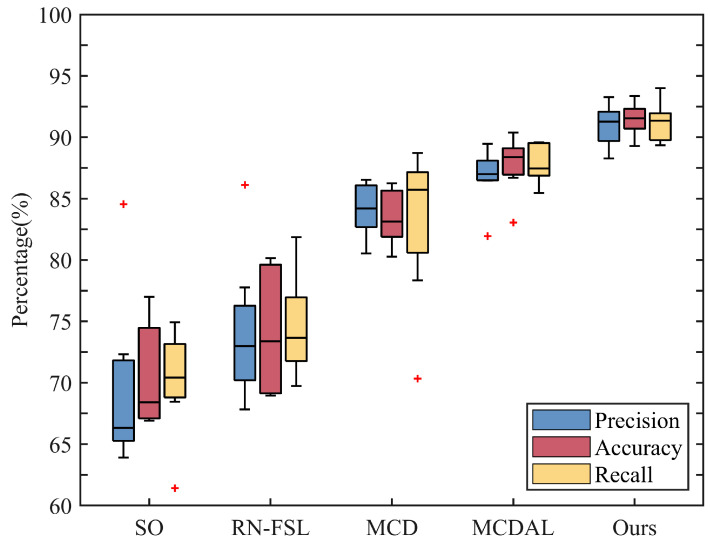
Comparison of precision, accuracy, and recall across five methods on the ENABL3S dataset. In the box plot, the median is represented by the horizontal line, and the box extends from the 25th to the 75th percentile.

**(2)**  **Cross-subject Performance Evaluation:** Figure 5 illustrates a performance comparison of cross-subject methods based on the ENABL3S dataset. Prior to model transfer, the SO model, which was trained using only source-domain data, achieves an average cross-subject accuracy of only 68.41%, indicating significant differences in data distribution across domains. Upon applying transfer methods, there is a notable improvement in cross-subject performance for RN-FSL, MCD, MCDAL, and our method, with average recognition accuracies reaching 73.38%, 83.13%, 88.38%, and 91.54%, respectively. It is worth noting that the increases in precision (from 66.32% to 72.99%) and recall (from 70.42% to 73.66%) for RN-FSL over SO further highlights the effectiveness of the relation network architecture in mitigating domain differences. Furthermore, compared to other cross-subject methods, our method demonstrates superior and more stable performance in precision (91.27%), accuracy (91.54%), and recall (from 87.46% to 91.34%) metrics, establishing the superiority of our approach.

**Figure 6:**
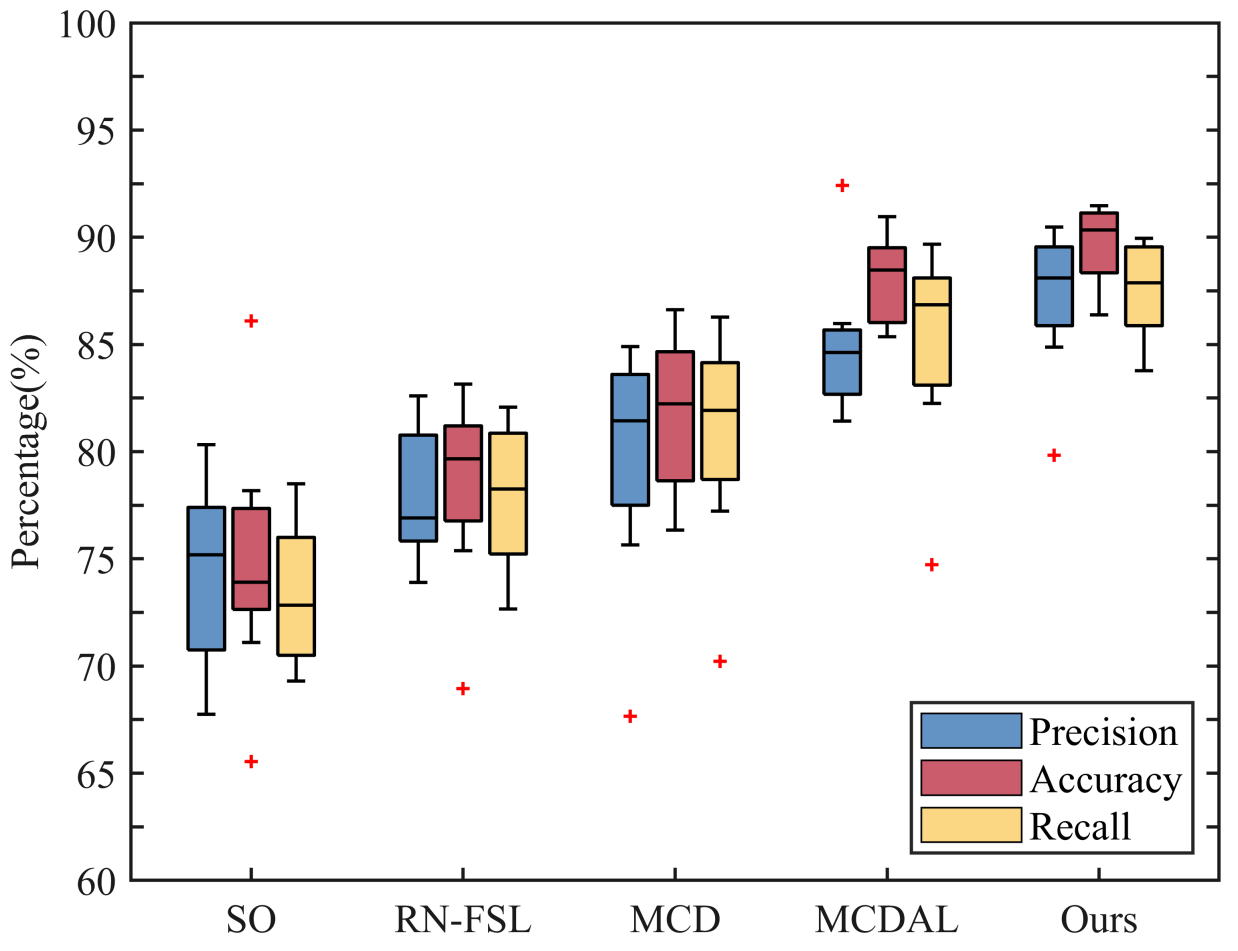
Comparison of precision, accuracy, and recall across five methods on our EMG dataset.

Figure 6 illustrates a performance comparison of different transfer methods based on our self-constructed EMG dataset. Prior to model transfer, the average cross-subject accuracy of SO is 73.91%. Following the application of model transfer, we observe notable improvements in accuracy rates for RN-FSL, MCD, MCDAL, and our method, recording values 79.67%, 82.23%, 88.47%, and 90.34%, respectively. All methods substantially enhance the cross-subject model performance. Our framework demonstrates a marked increase in both precision (from 75.19% to 88.10%) and recall (from 72.84% to 87.88%) post model transfer, affirming the effectiveness of our method. When directly compared to MCDAL, our method exhibits further improvements in precision (from 84.63% to 88.10%) and recall (from 86.85% to 87.88%), underscoring the critical role of updating the template buffer in optimizing performance. Among these methods, our proposed method performs best across all three statistical metrics evaluated in this subsection, thereby demonstrating the feasibility of our method.

**Figure 7:**
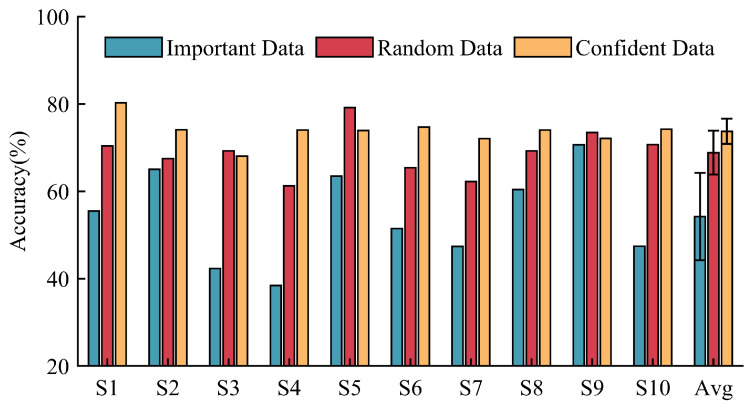
Comparison of recognition accuracy on target subjects using different template samples based on the ENABL3S dataset.

**(3)**  **Ablation Experiments:** We initially conducted experiments to assess the impact of different template sample types on model performance. Two types of sampled data, as well as random data, were utilized as template samples to directly evaluate the performance of the non-transferred relation model on new users. As depicted in Figure 7, we analyzed the influence of template selection methods on model performance based on the ENABL3S dataset. When important data served as templates, the average cross-subject accuracy of the model was only 54.18%, with notable variance. In contrast, utilizing random data and confident data as templates leads to a significant improvement in the model’s recognition accuracy for each new subject, with average recognition accuracies reaching 68.83% and 73.71%, respectively. This highlights the fact that the quality of template samples has a direct impact on both the performance and stability of the model.

**Figure 8:**
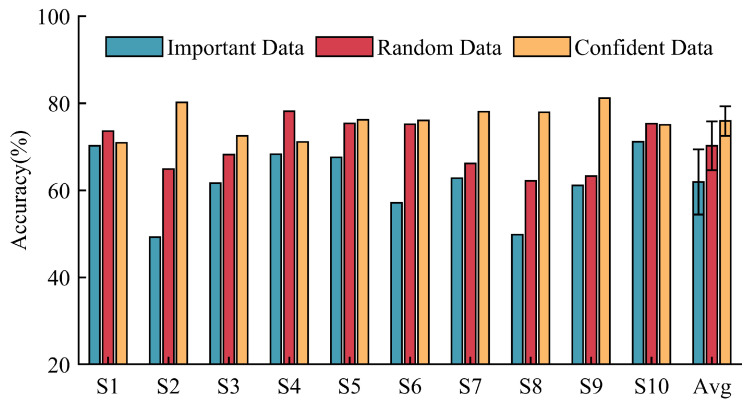
Comparison of recognition accuracy on target subjects using different template samples based on our EMG dataset.

Figure 8 illustrates the impact of template sample types on cross-subject model performance based on our self-constructed EMG dataset. The model utilizing important data as template samples achieves an average cross-subject recognition accuracy of 61.88%, with the lowest recognition accuracy of 49.21% observed for subject S2. In contrast, models using random data and confident data as template samples achieve average recognition accuracies of 70.11% and 74.40%, respectively. These two methods significantly enhance the cross-subject performance of the model.

**Figure 9:**
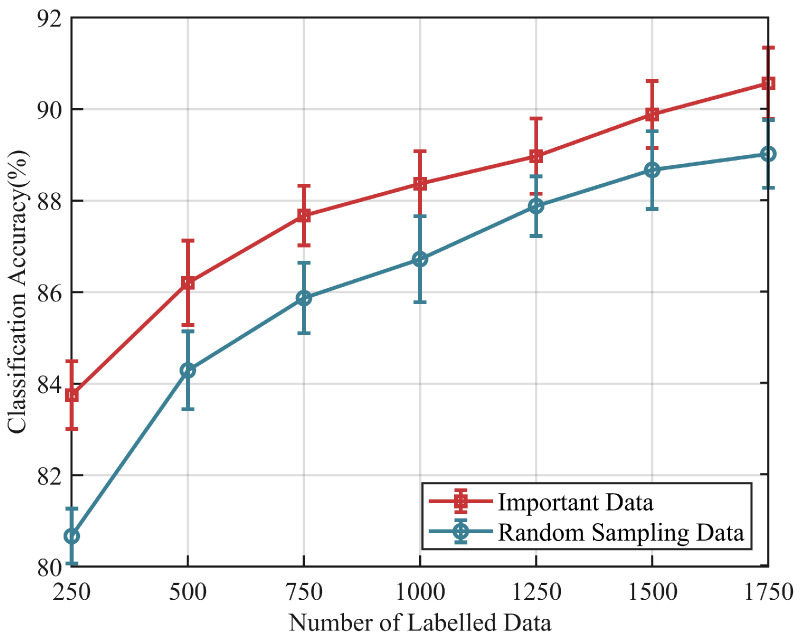
Effect of the new subject sample size on the classification accuracy using two different training data samples based on the ENABL3S dataset.

Subsequently, we evaluated the impact of varying quantities of two types of training samples on the model’s classification accuracy. Figure 9 shows the effect of training sample selection on model performance based on the ENABL3S dataset. The model utilizing random sampling data achieves an accuracy of 80.67% with 250 labeled samples and 89.02% with 1750 labeled samples. In contrast, the model based on important data consistently outperforms throughout, achieving an average classification accuracy of 90.56% with 1750 labeled samples and still exhibiting an upward trend.

**Figure 10:**
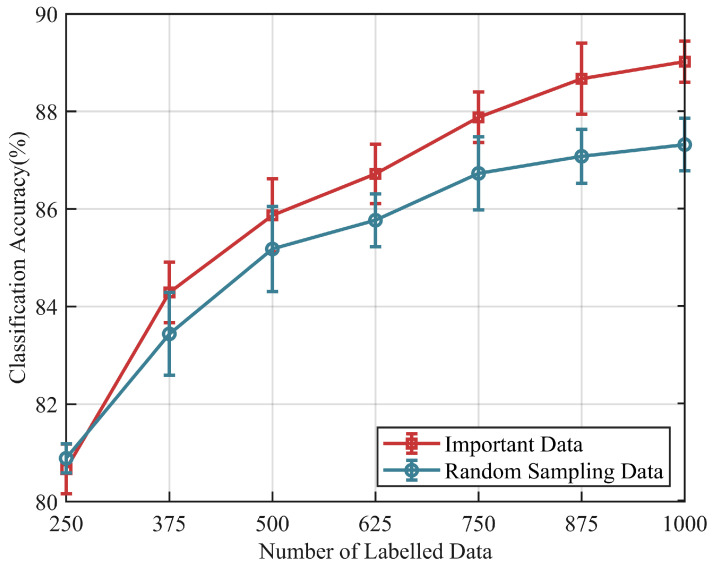
Effect of the new subject sample size on the classification accuracy using two different training data samples based on our EMG dataset.

The validation results of training samples on our EMG dataset are shown in Figure 10. Compared to the model trained with the random sampling data, the model based on the “important data” exhibits superior performance, which is particularly evident in the later stages. In our experimental setup, using 2000 labeled data samples achieves an accuracy of 90.34%, while our method achieves the highest performance of 89.14% with only 1000 labeled data. It is noteworthy that the accuracy of the model trained with randomly sampled data begins to saturate in the later stages, while our model still shows an upward trend.

In essence, the ablation studies not only substantiate the necessity of diligent screening of template samples and training samples but also showcases the incremental benefits these strategies contribute to the overall framework’s precision and reliability in motion-intent perception analysis.

## 5. Discussion

In this paper, an active learning framework that combines the relation network architecture with data sampling is proposed. This framework facilitates model adaptation to a new user by optimizing the model using a dual strategy. We aimed to construct a template set and a sample set for the target domain based on classifier discrepancy to facilitate the trained model in quickly adapting to the target domain and being competitive. We validated the effectiveness of our method by comparing it with other methods on EMG datasets. Furthermore, we emphasize the necessity of screening template samples and training samples by comparing the impacts of different data selection approaches on model performance.

**Distinctive benefits of the relation network architecture:** Compared to traditional network architectures with a single feature space, the relation network architecture markedly enhances the model’s generalization performance. By expanding the similarity space between the input sample and the template, this unique architecture captures both the core features of an individual sample and the shared features among different samples. The relation network architecture enhances the stability of the model by utilizing the similarity between the input sample and the template for motion prediction. Experimental results show that models employing the relation network architecture outperform the initial baseline model across all three statistical metrics (Figure 5 and Figure 6), highlighting its clear advantages in the field of HAR. Additionally, inspired by the dual-input samples of the network, we discovered that adjusting the number of templates or the data distribution of templates can impact the model’s performance. Considering both classification accuracy and testing time, the five-shot scenario stands out with its high performance and moderate time cost (Table 2). As more templates are added, the model can capture the features of samples with varying movements, muscle activities, and transition phases. However, an excessive number of template samples significantly increases the computational burden of the model. The impact of template data distribution on the model is in detail later on.

**Superior cross-subject performance of our framework:** The bidirectional optimization strategy significantly enhances the cross-subject performance of the model (Figure 5 and Figure 6). It utilizes both labeled target-domain data for backpropagation to adjust model parameters and the clustering algorithm to optimize the template input of the model, greatly improving the model’s stability and classification performance. By forward optimizing the template data distribution, the model becomes capable of capturing diverse representative features. Even within the same class of template data, the model can capture different features to better compare the similarity between the input sample and the template. Compared to before model transfer, both MCD and MCDAL improve the model’s cross-subject performance. These two methods adjust the feature space and classification hyperplane using unlabeled or labeled data for backpropagation, which greatly improves the cross-subject performance of the models. However, MCD exhibits lower accuracy, possibly due to the biased supervision information extracted from domain-intrinsic structures. Compared to MCDAL, our method demonstrates slight improvements across all three metrics and exhibits greater performance stability. This validates the feasibility of optimizing the distribution of template data.

**Necessity of data screening:** Data screening can achieve better performance based on a fixed number of samples. Based on classifier discrepancy, we filter target data into “confident data” and “important data”. Confident data, situated near the center of the domain, possess more representative features and are better-suited as template data for the relation network architecture. This aids the model in extracting domain-invariant features. The model using “important data” as template samples exhibits low performance and high variance (Figure 7 and Figure 8). This is because “important data” are far from the domain center and lack significant features. Additionally, these samples may be in transitional phases of movement, lacking the core features of independent class samples. Conversely, models using confident data as template samples demonstrate superior cross-subject performance, confirming the importance of template samples in maintaining model performance and stability. Secondly, “important data”, near the decision boundary and challenging for the model to identify, contribute to a tighter classification hyperplane when included in the training process. Compared to the model trained with randomly sampled data, the model adjusted with “important data” exhibits higher classification accuracy and shows the best performance gain in the final stages (Figure 9 and Figure 10). It is evident that random sampling data are not always helpful to the model.

In summary, the relation network architecture introduces a unique similarity space, enhancing the generalization performance of the model. Drawing inspiration from dual-input samples, our method substantially improves the model’s cross-subject performance by backward adjusting the model parameters and forward optimizing the template data distribution. Furthermore, “confident data” and “important data” obtained based on classifier discrepancy make an important contribution to the model adaptation process.

## 6. Conclusions

We propose a novel active learning framework based on the relation network architecture to address the cross-subject issue in HAR tasks. By filtering target data into “important data” and “confident data” based on classifier discrepancy, our method combines the importance of different data to enhance the efficiency of data utilization by the deep network. Additionally, we introduce a novel bidirectional optimization strategy to update the model parameters and optimize the template data distribution, effectively improving the model’s stability and cross-subject performance. We evaluated the generality of the proposed framework using both a public dataset and a self-built dataset, comparing multiple methods to demonstrate the validity and superiority of our method. Comparative experiments proved that our method achieves excellent cross-subject performance while maintaining model stability. Furthermore, ablation experiments demonstrated the necessity of reasonably screening templates samples and training samples during the model adaptation process. From the data perspective, the framework provides an effective solution to the cross-subject issue and contributes to a better application of HAR methods in exoskeleton control systems.

## Figures and Tables

**Table 1:** List of notations and descriptions utilized in this study.

Notation	Description
$D_{U}=\left\{x_{u}\right\}$	Target dataset
$D_{L}=\left\{\left(x_{l},y_{l}\right)\right\}$	Source dataset
$S=\left\{\left(x_{s},y_{s}\right)\right\}$	Sample set
$T=\left\{\left(x_{t},y_{t}\right)\right\}$	Template set
$G_{\phi }\left(\cdot \right)$	Feature extractor
$F_{\theta }\left(\cdot \right)$	Main classifier
$F_{\theta 1}\left(\cdot \right)\;\&\;F_{\theta 2}\left(\cdot \right)$	Auxiliary classifiers
$M\left(\cdot ,\cdot \right)$	Feature combination operator
$r\left(\left.y\right|x\right)$	Relation score from the main classifier
$r_{1}\left(\left.y\right|x\right)\;\&\;r_{2}\left(\left.y\right|x\right)$	Relation scores from the auxiliary classifiers
S(·)	Acquisition function
D(x)	Total discrepancy metric given a sample

**Table 2:** The classification accuracies and average testing time comparison of 1-shot, 5-shot, and 10-shot scenarios with new subjects based on the ENABL3S dataset and our EMG dataset.

Subject	7-Way Accuracy (%)			4-Way Accuracy (%)		
	1-Shot	5-Shot	10-Shot	1-Shot	5-Shot	10-Shot
Subject 4	60.17	80.15	81.93	66.71	76.50	79.51
Subject 5	39.28	73.38	76.47	73.55	81.17	85.06
Subject 6	48.54	79.43	83.31	68.41	77.73	82.66
Subject 7	61.33	69.20	71.36	63.23	80.43	83.24
Subject 8	58.45	68.95	75.65	70.40	79.62	84.23
Average Time	3.29 ms	4.95 ms	7.07 ms	1.94 ms	2.87 ms	4.98 ms

## Data Availability

The data presented in this study are available upon request from the corresponding author.
